# Similarities and Differences between Three-Dimensional Speckle-Tracking Echocardiography-Derived Left and Right Atrial Volumes and Volume-Based Functional Properties in the Same Healthy Adults—A Detailed Analysis from the MAGYAR-Healthy Study

**DOI:** 10.3390/medicina59122051

**Published:** 2023-11-21

**Authors:** Attila Nemes, Árpád Kormányos, Nóra Ambrus, Csaba Lengyel

**Affiliations:** Department of Medicine, Albert Szent-Györgyi Medical School, University of Szeged, 6725 Szeged, Hungary; kormanyos.arpad@med.u-szeged.hu (Á.K.); ambrus.nora@med.u-szeged.hu (N.A.); lengyel.csaba@med.u-szeged.hu (C.L.)

**Keywords:** left, right, atrial, volume, three-dimensional, echocardiography, function

## Abstract

*Background and Objectives*: It would be important to know what happens to the volume and volume-based functional properties of one atrium if the size of the other atrium is larger or smaller than the average. Therefore, the present study aimed to perform three-dimensional speckle-tracking echocardiography (3DSTE)-derived quantification of left atrial (LA) and right atrial (RA) volumes and volume-based functional properties to examine these associations in healthy adults with mean and lower or higher than mean atrial volumes. *Materials and Methods*: The present study consisted of 179 healthy volunteers with a mean age of 32.3 ± 12.3 years (92 males). Three-dimensional speckle-tracking echocardiography-derived LA and RA volumes and volume-based functional properties were determined in all cases. *Results*: When different LA or RA volume groups were evaluated, both LA and RA showed the same pattern of volume changes in all phases of atrial function with higher LA or RA volumes. In case of low and mean LA volumes, RA volumes were higher compared to their LA counterpart. In case of mean and high RA volumes, RA volumes proved to be higher as well. In case of mean LA or RA volumes, differences between LA and RA stroke volumes (SVs) could not be detected, but all atrial emptying fractions (EFs) were lower for RA than for LA. Some differences were detected in counterpart LA/RA total, passive, and active atrial SVs and EFs values in the presence of lower/higher than mean LA/RA volume. *Conclusions*: In case of mean LA or RA volumes, RA volumes are higher compared to their LA counterpart, LA-SVs and RA-SVs are similar, but atrial EFs are lower for RA than for LA. If lower/higher than mean LA or RA volumes are present, some differences in patterns of changes in counterpart atrial volumes—SVs and EFs—could be detected.

## 1. Introduction

Due to outstanding developments in echocardiography, detailed volumetric and functional analysis of heart chambers can be performed non-invasively in a relatively short time. The most recent three-dimensional (3D) speckle-tracking echocardiography (STE) with virtually created digital models can perform detailed volumetric and functional analysis of both atria at the same time using focused 3D echocardiographic datasets [1,2,3,4]. It is known that the left (LA) and right (RA) atria have complex functions during the cardiac cycle [5,6]. Although 3DSTE-derived normal reference values of LA and RA are known, and dimensions of both atria determined by other methods differ, it would be important to know what happens to the volume and volume-based functional properties of one atrium if the size of the other atrium is larger or smaller than the average [7,8,9]. Knowing this is essential for clinicians, as many medical conditions are associated with volumetric and functional variations in the heart chambers. Moreover, in addition to the difficulty of routine testing for RA, it can show early enlargement in many pathological conditions such as pulmonary disorders, congenital heart diseases, acquired valvular diseases, and heart failure [9]. Therefore, the present study used 3DSTE-derived quantification of LA and RA volumes and volume-based functional properties to examine these associations in healthy subjects with mean and lower or higher than mean atrial volumes.

## 2. Materials and Methods

### 2.1. Subjects

The present study consisted of 179 healthy volunteers with a mean age of 32.3 ± 12.3 years (92 males). All subjects participated in the study voluntarily between 2011–2015, and a physical examination, laboratory test, standard 12-lead electrocardiography (ECG), and two-dimensional (2D) Doppler echocardiography were performed with a negative result. The inclusion criteria included: non-smoker, non-obese (body mass index < 30 kg/m^2^), not taking any medication, and having no known medical conditions. Three-dimensional speckle-tracking echocardiography-derived data acquisitions were done immediately after complete 2D echocardiography, according to recent guidelines and practices. The present retrospective cohort study is part of the ‘Motion Analysis of the heart and Great vessels bY three-dimensionAl speckle-tRacking echocardiography in Healthy subjects’ (MAGYAR-Healthy) Study, which was organized at the University of Szeged for the purpose of carrying out physiological clinical studies (‘Magyar’ means ‘Hungarian’ in Hungarian language) [1,8,10,11,12]. The study was conducted in accordance with the Declaration of Helsinki (as revised in 2013). The Institutional and Regional Human Biomedical Research Committee of the University of Szeged, Hungary (No.: 71/2011), approved the study and all subjects gave informed consent.

### 2.2. Two-Dimensional Doppler Echocardiography

To perform 2D echocardiographic studies, a Toshiba Artida^TM^ echocardiographic device (Toshiba Medical Systems, Tokyo, Japan) attached to a 1–5 MHz PST-30BT phased-array transducer was used. Two-dimensional echocardiographic examinations were performed in accordance with professional guidelines and practices. First of all, subjects were asked to lie on their left side, then the transducer was placed on their chest and measurements were carried out from typical views. Following the measurement of chamber dimensions, derived volumetric assessments, and determination of left ventricular (LV) ejection fraction using the Simpson’s formula were performed, and valvular stenoses and regurgitations on any valves were excluded by Doppler measurements [13,14]. Early (E) and late (A) diastolic transmitral flow velocities (and their ratio) for the LV diastolic function were measured via pulsed Doppler.

### 2.3. Three-Dimensional Speckle-Tracking Echocardiography

The same Toshiba Artida^TM^ cardiac ultrasound device (Toshiba Medical Systems, Tokyo, Japan) was used after replacing the transducer with a PST-25SX matrix transducer. In the first phase of the study, 3D echocardiographic datasets were collected from the apical window by the same observer (ÁK). The patient in sinus rhythm was asked to lie on their left side, to hold his/her breath in inspiration, and to not move. During data acquisition, 6 subvolumes were acquired within 6 cardiac cycles following image optimizations, which were merged together automatically for a full-volume 3D echocardiographic dataset by the software. LA-, LV- and RA-focused datasets were acquired one after the other. 

### 2.4. Three-Dimensional Speckle-Tracking Echocardiography-Derived Atrial Quantifications

**Figure 1:**
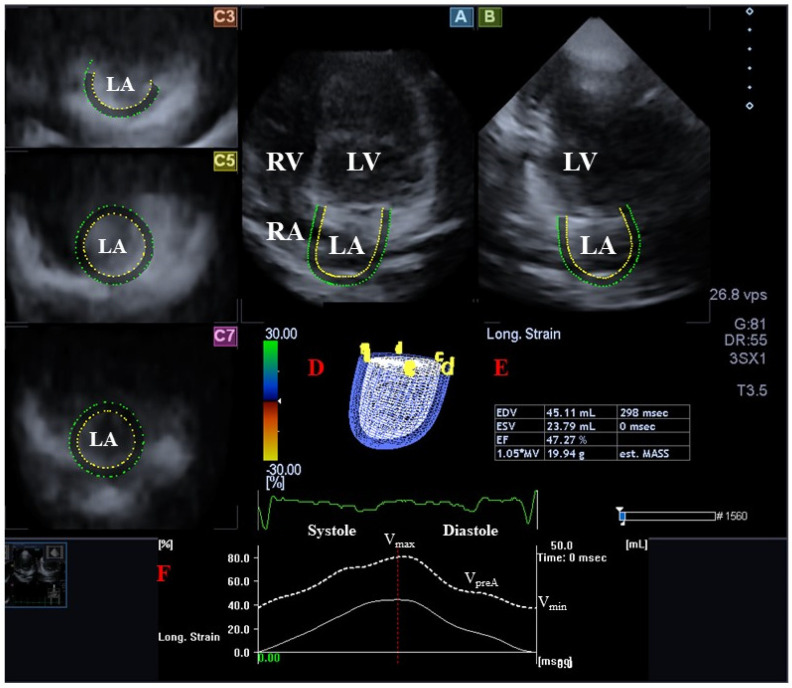
Assessment of the left atrium (LA) via three-dimensional (3D) speckle-tracking echocardiography using apical longitudinal four-chamber (**A**) and two-chamber (**B**) views, and short-axis views at basal (**C3**), midatrial (**C5**), and superior LA level (**C7**). Three-dimensional model of the LA (**D**), calculated LA volumetric data (**E**), and time— LA global volume changes (white dotted line) during the cardiac cycle are also presented (**F**) together with maximum (V_max_), preatrial contraction (V_preA_), and minimum (V_min_) LA volumes. Abbreviations: LA, left atrium; LV, left ventricle; RA, right atrium; RV, right ventricle; EDV, end-diastolic volume; ESV, end-systolic volume; EF, ejection fraction; V_max_, end-systolic maximum LA volume; V_preA_, early diastolic pre-atrial contraction LA volume; V_min_, end-diastolic minimum LA volume.

**Figure 2:**
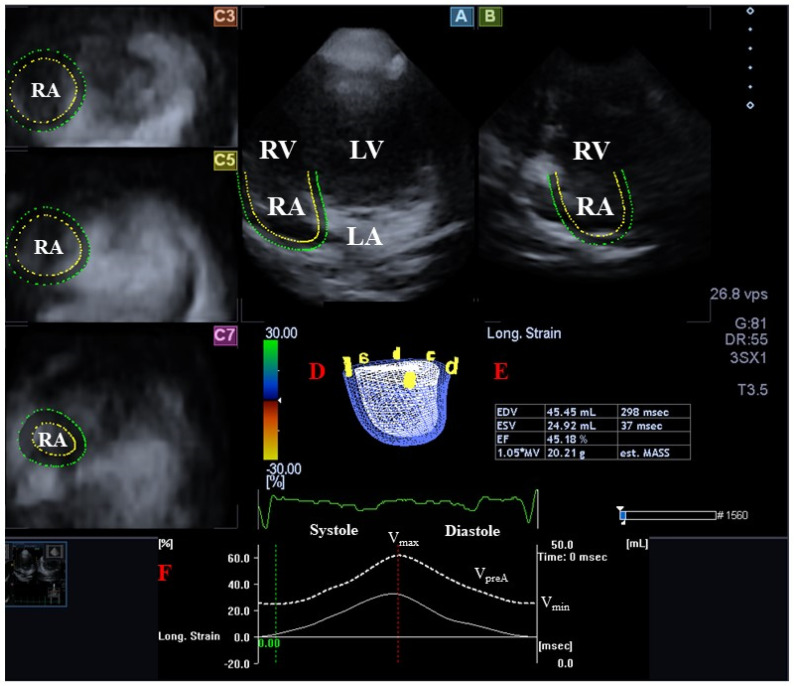
Assessment of the right atrium (RA) via three-dimensional (3D) speckle-tracking echocardiography using apical longitudinal four-chamber (**A**) and two-chamber (**B**) views, and short-axis views at basal (**C3**), midatrial (**C5**), and superior RA level (**C7**). Three-dimensional model of the RA (**D**), calculated RA volumetric data (**E**), and time— RA global volume change (white dotted line) during the cardiac cycle are also presented (**F**) together with maximum (V_max_), preatrial contraction (V_preA_), and minimum (V_min_) RA volumes. Abbreviations: LA, left atrium; LV, left ventricle; RA, right atrium; RV, right ventricle; EDV, end-diastolic volume; ESV, end-systolic volume; EF, ejection fraction; V_max_, end-systolic maximum RA volume; V_preA_, early diastolic pre-atrial contraction RA volume; V_min_, end-diastolic minimum RA volume.

Following LA/RA-focused data acquisitions, offline analysis was performed with the vendor-provided 3D Wall Motion Tracking software version 2.7 (Ultra Extend, Toshiba Medical Systems, Tokyo, Japan) at a later date [1,2,3,4]. The data were presented on automatically generated end-diastolic images in select apical two- (AP2CH) and four-chamber (AP4CH) views and 3 short-axis views at the basal, midatrial, and superior levels. Reference points were defined on the LA/RA endocardium to create digital models of the LA/RA in AP2CH and AP4CH views following the definition of the edges of the RA-tricuspid annulus, the LA-mitral annulus rings, and the LA/RA apices at end-diastole. Automatic sequential analysis (reconstruction) was performed for the complete endocardial LA/RA surface. The following LA/RA volumes have been calculated, taking into account the heart cycle [7,8,10,11] (Figure 1 and Figure 2):

- V_max_—maximum LA/RA volume measured at end-systole (just before mitral/tricuspid valve opening).
- V_preA_—LA/RA volume before atrial contraction measured at early-diastole (at the time of the P wave on the ECG).
- V_min_—minimum LA/RA volume measured at end-diastole (just before mitral/tricuspid valve closure).

The following LA/RA stroke volumes (SV) and emptying fractions (EF) were assessed:

- For characterization of LA/RA reservoir function:
  ∘ Total atrial stroke volume (TASV): V_max_ − V_min_.
  ∘ Total atrial emptying fraction (TAEF): TASV/V_max_ × 100.
- For characterization of LA/RA conduit function:
  ∘ Passive atrial stroke volume (PASV): V_max_ − V_preA_.
  ∘ Passive atrial emptying fraction (PAEF): PASV/V_max_ × 100.
- For characterization of LA/RA active contraction:
  ∘ Active atrial stroke volume (AASV): V_preA_ − V_min_.
  ∘ Active atrial emptying fraction (AAEF): AASV/V_preA_ × 100.

In each case, a number of measurements were carried out, and if the measurement results using the 3D echocardiographic datasets proved to be consistent, they were accepted.

### 2.5. Statistical Analysis

The mean ± standard deviation (SD) format was used for continuous variables, and the number/percentage format was used for categorical variables. *p* less than 0.05 was considered to be statistically significant. Independent sample *t*-tests and analysis of variance (ANOVA) tests were performed for group comparisons where appropriate. To determine intraobserver and interobserver agreements, the Bland–Altman method was used in 30 randomly selected healthy subjects; for intraobserver and interobserver correlations, intraclass correlation coefficients (ICCs) were calculated. SPSS software version 22 was used for statistical analyses (SPSS Inc., Chicago, IL, USA).

## 3. Results

### 3.1. Clinical and Two-Dimensional Doppler Echocardiographic Data

Mean systolic and diastolic blood pressure, heart rate, height, and weight were 121.3 ± 4.2 mm Hg, 77.3 ± 2.7 mm Hg, 71.5 ± 1.8 1/s, 172.3 ± 9.9 cm, and 69.7 ± 14.2 kg, respectively. All the routine 2D echocardiographic data were in normal ranges, including LA diameter measured in parasternal long-axis view, LV end-diastolic diameter and volume, LV end-systolic diameter and volume, interventricular septum, LV posterior wall, and LV ejection fraction which proved to be 36.5 ± 4.0 mm, 48.0 ± 3.7 mm, 106.2 ± 23.0 mL, 31.8 ± 3.1 mm, 36.2 ± 8.8 mL, 8.9 ± 1.5 mm, 9.1 ± 1.6 mm, and 65.9 ± 4.7%, respectively. Mean E/A proved to be 1.35 ± 0.39. None of the healthy subjects demonstrated larger than grade 1 valvular regurgitation or significant stenosis on any valves.

### 3.2. Classification of Subjects

Mean ± SD of 3DSTE-derived LA and RA volumes and volume-based functional properties of healthy cases are presented in Table 1. Healthy subjects were classified into three groups according to the normal LA-V_max_, LA-V_preA_, LA-V_min_, RA-V_max_, RA-V_preA_, and RA-V_min_: estimated mean ± SD served as the lower (27.6 mL, 15.7 mL, 11.3 mL, 32.7 mL, 22.5 mL, and 16.4 mL, respectively) and upper (53.8 mL, 39.5 mL, 27.9 mL, 61.3 mL, 44.9 mL, and 36.0 mL, respectively) values.

### 3.3. Atrial Volumes in Different Left Atrial Volume Groups

When different LA volume groups were evaluated, both RA and LA showed the same pattern of volume changes in all phases of atrial function with higher LA volumes. When RA and LA volumes at the same phase of the cardiac cycle were compared, in the case of low and mean LA volumes, RA volumes were higher compared to their LA counterparts. In the case of high LA volumes, RA volumes were lower, except for RA-V_min_, when LA and RA volumes were similar. In case of high LA-V_min_, LA and RA volumes were also similar (Table 2 and Table 3).

### 3.4. Atrial Volumes in Different Right Atrial Volume Groups

When different RA volume groups were compared, both atria showed the same pattern of volume changes in all phases of atrial function with increased RA volumes. When RA and LA volumes at the same phase of cardiac cycle were compared, in the case of low RA volumes, LA and RA volumes were similar. In case of mean and high RA volumes, RA volumes proved to be higher compared to their LA counterparts (Table 4 and Table 5).

### 3.5. Atrial Stroke Volumes in Different Left Atrial Volume Groups

During the investigation of LA volume groups, changes in LA-TASV, LA-AASV (in the case of all higher LA volumes), and PASV (in the case of higher LV-V_max_) were present without significant changes in RA-TASV and RA-PASV. Only RA-AASV showed an increase in the case of all higher LA volumes (Table 2). When RA and LA stroke volumes at the same phase of the cardiac cycle were compared, in the case of low LA-V_max_, higher RA-SVs could be detected compared to LA-SVs. In the case of low LA-V_preA_, TASVs and PASVs were similar, and only RA-AASV was higher compared to LA-AASV. In the case of low LA-V_min_, LA-SVs and RA-SVs were similar. In the case of high LA volumes, RA-TASV and RA-AASV were lower, but a similar RA-PASV could be detected compared to their LA counterpart. In the case of mean LA volumes, LA-SVs and RA-SVs were similar (Table 2 and Table 3).

### 3.6. Atrial Stroke Volumes in Different Right Atrial Volume Groups

During the investigation of RA volume groups, both RA and LA showed same pattern of atrial SV changes with increased RA volumes, except for AASV being more pronounced in the case of the LA. When the RA-SVs and LA-SVs at the same phase of the cardiac cycle were compared, in the case of low RA-V_max_, lower RA-SVs were present compared to LA-SVs. In the case of low RA-V_preA_ and RA-V_min_, LA-SVs and RA-SVs were similar. In the case of high RA-V_max_, higher RA-TASV and RA-PASV, but similar RA-AASV could be detected compared to their LA counterpart. In the case of high RA-V_preA_, LA-SVs and RA-SVs were similar. In the case of high RA-V_min_, LA-SVs and RA-SVs were similar—except for AASV, which was lower for RA. In case of mean RA volumes, LA-SVs and RA-SVs were similar (Table 4 and Table 5).

### 3.7. Atrial Emptying Fractions in Different Left Atrial Volume Groups

When different LA volume groups were evaluated, TAEF in the LA-V_max_ group and AAEF in all LA volume groups showed a different pattern of changes. When RA-EFs and LA-EFs at the same phase of the cardiac cycle were compared, in the case of low LA-V_max_, similar LA-EFs and RA-EFs could be detected. In the case of low LA-V_preA_, lower RA-TAEF and RA-PAEF with similar RA-AAEF could be demonstrated when they were compared to their LA counterpart. In the case of low LA-V_min_, all atrial EFs were lower for RA compared to their LA counterpart. In the case of high LA volumes, TAEF and AAEF were lower, with a preserved PAEF for RA compared to LA. In the case of mean LA volumes, all EFs were lower for RA than for LA (Table 2 and Table 3).

### 3.8. Atrial Emptying Fractions in Different Right Atrial Volume Groups

PAEF in RA-V_max_ groups and AAEF in RA-V_preA_ and RA-V_min_ groups showed a different pattern of changes with increased RA volumes. When RA-EFs and LA-EFs at the same phase of the cardiac cycle were examined, in the case of low RA-V_max_, all EFs were lower for RA compared to LA. In the case of low RA-V_preA_ and RA-V_min_, RA-EFs did not differ compared to LA-EFs. In the case of high RA-V_max_, only the AAEF differed, with similar TAEF and PAEF for both atria. In the case of high RA-V_preA_ and RA-V_min_, all RA-EFs were lower compared to all LA-EFs. In the case of mean RA volumes, all EFs were lower for RA than for LA (Table 4 and Table 5).

### 3.9. Reproducibility of Three-Dimensional Speckle-Tracking Echocardiography-Derived Left Atrial/Right Atrial Assessments

Three-dimensional speckle-tracking echocardiography-derived LA and RA volumes were measured twice by the same observer (intraobserver agreement) and by two independent observers (interobserver agreement). The values were expressed as mean ± SD together with corresponding ICCs, and the results are presented in Table 6.

## 4. Discussion

In recent decades, there have been huge technical advances in cardiovascular imaging. Not only is the spread of cardiac magnetic resonance imaging and computer tomography observed, but new echocardiographic procedures have appeared as well. STE is one of the most recent developments, which can see and visualize the heart and its cavities, including both atria, using 3D models [7,8,10,11]. Three-dimensional speckle-tracking echocardiography is suitable for detailed atrial volumetric and volume-based functional analysis using SVs and EFs, respecting the cardiac cycle [7,8,10,11]. Three-dimensional speckle-tracking echocardiography is not only validated for atrial measurements [12,15,16]; 3DSTE-derived normal reference values for LA and RA volumes are also available [7,8].

The atria not only differ in shape, but also in that while the LA fills from the four pulmonary veins and empties through the mitral valve towards the LV, the RA fills from both the caval veins and the sinus coronarius and empties into the RV through the tricuspid valve. Their functions similarly include a reservoir phase in systole, a conduit phase in early diastole, and a booster pump phase in late diastole [5,6]. In recent clinical studies, several differences could be demonstrated between atrial functions. For instance, similarly to the LV, the Frank–Starling mechanism could be demonstrated in the LA [17,18]. Increased LA strains were seen in healthy subjects with greater LA volumes, but only up to a point beyond which this association disappeared [10]. Interestingly, similar obvious associations for the RA could not be detected [11]. Moreover, different behaviour between LA and RA strains were seen in recent 3DSTE studies, together with gender-dependency of RA strains, which were not present for LA strains [8,9].

The present clinico-physiological study tried to extend our knowledge to investigate what happens to the volumes, SVs, and EFs of one atrium when the volume of the other atrium is lower or higher than the mean value in healthy adults. The main finding of the present study is that if mean LA or RA volumes are present, RA volumes are higher compared to their LA counterpart. With detailed analyses presented above, atrial SVs proved to be similar for both atria, but atrial EFs were lower for the RA compared to that of the LA in the case of mean atrial volumes. Some different patterns of changes in atrial volumes, SVs, and EFs could be detected if a LA or RA volume lower/higher than the mean was present. The interesting thing about the differences presented above is that they occurred in healthy subjects. The above results are only partially consistent with those previously described in a 2DSTE study by Moustafa et al. [19]. In this study, V_max_ for the LA proved to be higher than that of the RA with similar V_preA_ and V_min_. LA-TASV, LA-TAEF, and LA-PAEF were larger than that of the RA counterpart, with a similar AAEF. In our study, all RA volumes were larger and all EFs were lower for the RA than that of the LA counterpart. Differences can be explained by subject selections being in different ages (39 ± 15 years vs. 32 ± 12 years) and methodological differences (2D-STE vs. 3D-STE) [19].

Findings could be explained by the fact that the LA and RA are under different pressures, metabolic and flow conditions, and neural and mechanical regulations [20,21,22]. In recent studies examining certain pathologies, inter-chamber differences and asymmetrical remodeling of the LA and RA could be demonstrated [20,21,22]. Several studies have shown that both atria are dilated, and their function deteriorates with arrhythmological consequences in the presence of certain cardiac diseases, even in children [23,24,25]. Moreover, dilation of the atria could also be detected in different pathologies and elite athletes, whose cardiac consequences were not widely known until recent times [26,27]. Based on these findings, recent studies have raised the possibility that the combination of volumetric, volume-based, and strain atrial abnormalities may be disease-specific [11,12]. Therefore, further studies in different disorders are required for better understanding the effects of disease-related atrial volume changes on haemodynamics, myocardial mechanics, and vascular remodeling.

### Limitations

The following limitations should be taken into account when interpreting the results:

- If we compare the image quality of 2D echocardiography and 3DSTE, it can be concluded that the image quality of 2D echocardiography is still right due to the worse spatial and temporal resolution of 3DSTE [1,2,3,4].
- Three-dimensional speckle-tracking echocardiography is suitable for measuring so-called strain parameters simultaneously with volumetric measurements of both LA and RA, using the 3D models of the atria. However, this study did not aim to perform such assessments.
- Moreover, it did not aim to assess 3DSTE-derived LV/RV parameters either.
- The present study did not aim to validate 3DSTE-derived atrial parameters due to their validated nature [15,16].
- The atrial septum was part of the 3D model during the analysis of both atria, which can be considered a serious limitation.
- Calculation of body mass indexed atrial volumes would have been performed. However, the purpose of the present study was to examine hearts of different sizes and volumes, not individuals. Accordingly, body mass indexation has no relevance.
- We could not absolutely exclude subjects who had any latent non-diagnosed disorders, which could affect results.

## 5. Conclusions

In the case of mean LA or RA volumes, RA volumes are higher compared to their LA counterparts, LA-SVs and RA-SVs are similar, but atrial EFs are lower for the RA than for the LA. In the case of lower/higher than mean LA or RA volumes, some differences in patterns of changes in counterpart atrial volumes, SVs and EFs, could be detected.

## Figures and Tables

**Table 1:** Three-dimensional speckle-tracking echocardiography-derived atrial volumes and volume-based functional properties.

Data	Measures
Right atrium	
End-systolic maximum RA volume (V_max,_ mL)	47.0 ± 14.3 *
Early diastolic RA volume (V_preA_, mL)	33.7 ± 11.2 *
Late diastolic RA volume (V_min,_ mL)	26.2 ± 9.8 *
Total RA stroke volume (TASV, mL)	20.9 ± 9.3
Total RA emptying fraction (TAEF, %)	44.1 ± 13.2 *
Passive RA stroke volume (PASV, mL)	13.3 ± 7.8
Passive RA emptying fraction (PAEF, %)	27.9 ± 12.5 *
Active RA stroke volume (AASV, mL)	7.5 ± 4.6
Active RA emptying fraction (AAEF, %)	22.6 ± 11.6 *
Left atrium	
End-systolic maximum LA volume (V_max,_ mL)	40.7 ± 13.1
Early diastolic LA volume (V_preA_, mL)	27.6 ± 11.9
Late diastolic LA volume (V_min,_ mL)	19.6 ± 8.3
Total LA stroke volume (TASV, mL)	21.2 ± 8.0
Total LA emptying fraction (TAEF, %)	52.2 ± 12.0
Passive LA stroke volume (PASV, mL)	13.1 ± 5.6
Passive LA emptying fraction (PAEF, %)	33.4 ± 12.9
Active LA stroke volume (AASV, mL)	8.1 ± 5.5
Active LA emptying fraction (AAEF, %)	28.1 ± 11.7

Abbreviations: LA = left atrial, RA = right atrial; * *p* < 0.05 vs. LA counterpart.

**Table 2:** Right atrial volumes in different left atrial volume groups.

	LA-V_max_ < 27.6 mL (n = 26)	27.6 mL ≤ LA-V_max_ ≤ 53.8 mL (n = 124)	53.8 mL < LA-V_max_ (n = 29)	LA-V_preA_ < 15.7 mL (n = 25)	15.7 mL ≤ LA-V_preA_ ≤ 39.5 mL (n = 125)	39.5 mL < LA-V_preA_ (n = 29)	LA-V_min_ < 11.3 mL (n = 25)	11.3 mL ≤ LA-V_min_ ≤ 27.9 mL (n = 125)	27.9 mL < LA-V_min_ (n = 29)
V_max_ (mL)	35.7 ± 11.3 *	47.6 ± 13.5 *^†^	54.6 ± 13.9 *^†‡^	36.5 ± 11.0 *	47.1 ± 12.7 *^†^	55.0 ± 17.6 *^†‡^	36.6 ± 11.7 *	46.9 ± 12.8 *^†^	56.8 ± 15.7 ^†‡^
V_preA_ (mL)	23.3 ± 6.8 *	34.1 ± 10.3 *^†^	41.4 ± 10.8 *^†‡^	22.2 ± 6.3 *	33.6 ± 9.1 *^†^	43.2 ± 13.6 *^†‡^	23.3 ± 7.8 *	33.2 ± 9.0 *^†^	44.8 ± 12.3 ^†‡^
V_min_ (mL)	18.0 ± 5.4 *	26.3 ± 9.0 *^†^	32.8 ± 10.5 ^†‡^	17.2 ± 5.8 *	25.9 ± 8.4 *^†^	34.5 ± 11.3 ^†‡^	17.3 ± 6.3 *	25.8 ± 8.3 *^†^	35.7 ± 10.0 ^†‡^
TASV (mL)	17.7 ± 8.5 *	21.3 ± 9.2 ^†^	21.7 ± 10.1 *	19.3 ± 7.8	21.2 ± 9.2	20.6 ± 10.6 *	19.3 ± 8.3	21.1 ± 9.3	21.1 ± 10.3 *
TAEF (%)	48.2 ± 11.9	44.4 ± 12.7 *	39.2 ± 15.1 *^†‡^	52.2 ± 11.7 *	44.4 ± 12.6 *^†^	36.3 ± 13.0 *^†‡^	52.0 ± 12.6 *	44.4 ± 12.5 *^†^	36.1 ± 12.7 *^†‡^
PASV (mL)	12.4 ± 7.5 *	13.6 ± 7.4	13.1 ± 9.1	14.3 ± 6.8	13.5 ± 7.5	11.8 ± 9.4	13.3 ± 6.7	13.7 ± 7.6	12.0 ± 9.2
PAEF (%)	33.2 ± 13.6	28.0 ± 11.5 *^†^	22.7 ± 13.6 ^†‡^	38.1 ± 11.9 *	27.9 ± 11.3 *^†^	20.0 ± 12.6 ^†‡^	35.6 ± 13.7 *	28.3 ± 11.3 *^†^	19.9 ± 12.3 ^†‡^
AASV (mL)	5.3 ± 2.8 *	7.8 ± 4.5 ^†^	8.6 ± 5.6 *^†^	5.0 ± 2.4 *	7.7 ± 4.3 ^†^	8.8 ± 6.2 *^†^	6.0 ± 4.0	7.5 ± 4.4	9.1 ± 5.7 *^†^
AAEF (%)	22.4 ± 9.5	23.0 ± 11.5 *	21.3 ± 13.4 *	23.1 ± 10.3	23.1 ± 11.6 *	20.1 ± 12.2 *	25.5 ± 12.1 *	22.5 ± 11.5 *	20.0 ± 10.6 *

Abbreviations: V_max_ = end-systolic maximum volume, V_preA_ = early diastolic preatrial contraction volume, V_min_ = late diastolic minimum volume, TASV = total atrial stroke volume, TAEF = total atrial emptying fraction, PASV = passive atrial stroke volume, PAEF = passive atrial emptying fraction, AASV = active atrial stroke volume, AAEF = active atrial emptying fraction, RA = right atrial, LA = left atrial. * *p* < 0.05 vs. LA counterpart (Table 3); ^†^ *p* < 0.05 vs. lower than mean counterpart; ^‡^ *p* < 0.05 vs. mean counterpart.

**Table 3:** Left atrial volumes in different left atrial volume groups.

	LA-V_max_ < 27.6 mL (n = 26)	27.6 mL ≤ LA-V_max_ ≤ 53.8 mL (n = 124)	53.8 mL < LA-V_max_ (n = 29)	LA-V_preA_ < 15.7 mL (n = 25)	15.7 mL ≤ LA-V_preA_ ≤ 39.5 mL (n = 125)	39.5 mL < LA-V_preA_ (n = 29)	LA-V_min_ < 11.3 mL (n = 25)	11.3 mL ≤ LA-V_min_ ≤ 27.9 mL (n = 125)	27.9 mL < LA-V_min_ (n = 29)
V_max_ (mL)	24.0 ± 3.5	38.7 ± 6.4 ^†^	64.3 ± 6.8 ^†‡^	26.2 ± 5.4	38.5 ± 8.5 ^†^	62.4 ± 9.2 ^†‡^	27.0 ± 5.3	38.9 ± 9.1 ^†^	60.4 ± 10.5 ^†‡^
V_preA_ (mL)	15.3 ± 4.0	25.6 ± 7.0 ^†^	47.4 ± 10.4 ^†‡^	12.9 ± 2.3	25.3 ± 5.9 ^†^	50.0 ± 7.8 ^†‡^	14.0 ± 3.3	25.7 ± 7.1 ^†^	47.7 ± 8.9 ^†‡^
V_min_ (mL)	11.7 ± 3.5	18.3 ± 5.7 ^†^	31.9 ± 8.0 ^†‡^	9.6 ± 2.2	18.3 ± 5.0 ^†^	33.3 ± 6.9 ^†‡^	9.0 ± 1.6	18.3 ± 4.1 ^†^	34.3 ± 5.4 ^†‡^
TASV (mL)	12.2 ± 3.6	20.4 ± 5.4 ^†^	32.4 ± 7.3 ^†‡^	16.6 ± 5.2	20.2 ± 6.9 ^†^	29.1 ± 9.0 ^†‡^	18.0 ± 4.6	20.6 ± 7.8	26.1 ± 8.8 ^†‡^
TAEF (%)	50.9 ± 13.1	52.8 ± 12.0	50.6 ± 10.9	62.0 ± 10.0	51.8 ± 11.6 ^†^	46.1 ± 10.7 ^†‡^	65.6 ± 7.2	51.8 ± 10.9 ^†^	42.3 ± 9.1 ^†‡^
PASV (mL)	8.7 ± 3.4	13.1 ± 4.8 ^†^	16.9 ± 7.0 ^†‡^	13.3 ± 4.1	13.2 ± 5.7	12.6 ± 5.9	12.9 ± 4.3	13.2 ± 5.6	12.7 ± 6.3
PAEF (%)	36.3 ± 13.5	34.3 ± 12.4	26.7 ± 12.0 ^†‡^	50.0 ± 8.0	33.5 ± 11.0 ^†^	19.8 ± 7.9 ^†‡^	47.2 ± 10.9	33.7 ± 11.1 ^†^	20.6 ± 8.7 ^†‡^
AASV (mL)	3.6 ± 2.8	7.3 ± 3.5 ^†^	15.6 ± 7.2 ^†‡^	3.3 ± 1.9	7.1 ± 3.4 ^†^	16.5 ± 6.9 ^†‡^	5.0 ± 3.1	7.4 ± 4.9 ^†^	13.4 ± 6.3 ^†‡^
AAEF (%)	22.5 ± 14.3	28.3 ± 10.7 ^†^	32.3 ± 11.5 ^†^	25.0 ± 12.9	27.6 ± 11.3	32.7 ± 11.7 ^†‡^	33.3 14.0	28.2 ± 11.5 ^†^	27.2 ± 9.2 ^†^

Abbreviations: V_max_ = end-systolic maximum volume, V_preA_ = early diastolic preatrial contraction volume, V_min_ = late diastolic minimum volume, TASV = total atrial stroke volume, TAEF = total atrial emptying fraction, PASV = passive atrial stroke volume, PAEF = passive atrial emptying fraction, AASV = active atrial stroke volume, AAEF = active atrial emptying fraction, RA = right atrial, LA = left atrial. ^†^ *p* < 0.05 vs. lower than mean counterpart; ^‡^ *p* < 0.05 vs. mean counterpart

**Table 4:** Right atrial volumes in different right atrial volume groups.

	RA-V_max_ < 32.7 mL (n = 31)	32.7 mL ≤ RA-V_max_ ≤ 61.3 mL (n = 116)	61.3 mL < RA-V_max_ (n = 32)	RA-V_preA_ < 22.5 mL (n = 27)	22.5 mL ≤ RA-V_preA_ ≤ 44.9 mL (n = 130)	44.9 mL < RA-V_preA_ (n = 22)	RA-V_min_ < 16.4 mL (n = 27)	16.4 mL ≤ RA-V_min_ ≤ 36.0 mL (n = 122)	36.0 mL < RA-V_min_ (n = 30)
V_max_ (mL)	28.8 ± 3.0	45.5 ± 7.6 *^†^	71.0 ± 7.6 *^†‡^	30.4 ± 4.8	46.9 ± 11.0 *^†^	68.1 ± 11.3 *^†‡^	30.7 ± 6.3	46.6 ± 11.1 *^†^	63.4 ± 13.1 *^†‡^
V_preA_ (mL)	20.8 ± 4.8	33.1 ± 7.5 *^†^	47.9 ± 10.6 *^†‡^	18.3 ± 3.1	33.5 ± 6.2 *^†^	53.9 ± 8.6 *^†‡^	19.1 ± 4.3	32.8 ± 6.3 *^†^	50.5 ± 9.4 *^†‡^
V_min_ (mL)	16.2 ± 4.2	25.6 ± 7.6 *^†^	37.6 ± 8.6 *^†‡^	13.9 ± 3.0	25.7 ± 6.3 *^†^	43.9 ± 5.3 *^†‡^	13.2 ± 2.5	24.9 ± 5.0 *^†^	43.0 ± 4.5 *^†‡^
TASV (mL)	12.7 ± 3.5 *	19.9 ± 7.4 ^†^	33.4 ± 7.5 *^†‡^	16.6 ± 5.3	21.2 ± 9.5 †	24.2 ± 10.1 ^†^	17.5 ± 6.7	21.7 ± 9.3 ^†^	20.4 ± 10.6
TAEF (%)	44.2 ± 12.1 *	43.6 ± 13.9 *	47.2 ± 10.3	53.5 ± 11.6	43.8 ± 12.7 *^†^	34.3 ± 10.1 *^†‡^	55.4 ± 11.6	45.0 ± 11.0 *^†^	30.2 ± 10.9 *^†‡^
PASV (mL)	8.0 ± 4.0 *	12.3 ± 6.0 ^†^	23.1 ± 8.4 *^†‡^	12.1 ± 4.7	13.4 ± 8.0	14.2 ± 9.0	11.5 ± 5.2	13.8 ± 7.9	12.9 ± 8.88
PAEF (%)	28.0 ± 14.1 *	27.0 ± 11.8 *	32.6 ± 11.8 ^‡^	39.0 ± 11.4	27.0 ± 11.6 *^†^	20.0 ± 10.6 *^†‡^	36.7 ± 13.4	28.1 ± 11.3 *^†^	19.2 ± 10.6 *^†‡^
AASV (mL)	4.7 ± 2.1 *	7.5 ± 4.5 ^†^	10.3 ± 5.3 ^†‡^	4.4 ± 2.0	7.8 ± 4.5 ^†^	9.9 ± 5.8 ^†‡^	5.9 ± 3.4	7.9 ± 4.3 ^†^	7.5 ± 6.2 *
AAEF (%)	22.5 ± 8.5 *	23.1 ± 12.7 *	21.4 ± 9.2 *	24.4 ± 10.0	23.1 ± 12.1 *	17.7 ± 8.4 *^†‡^	29.6 ± 11.0	23.4 ± 10.6 *^†^	13.3 ± 10.0 *^†‡^

Abbreviations: V_max_ = end-systolic maximum volume, V_preA_ = early diastolic preatrial contraction volume, V_min_ = late diastolic minimum volume, TASV = total atrial stroke volume, TAEF = total atrial emptying fraction, PASV = passive atrial stroke volume, PAEF = passive atrial emptying fraction, AASV = active atrial stroke volume, AAEF = active atrial emptying fraction, RA = right atrial, LA = left atrial. * *p* < 0.05 vs. LA counterpart (Table 5); ^†^ *p* < 0.05 vs. lower than mean counterpart; ^‡^ *p* < 0.05 vs. mean counterpart.

**Table 5:** Left atrial volumes in different right atrial volume groups.

	RA-V_max_ < 32.7 mL (n = 31)	32.7 mL ≤ RA-V_max_ ≤ 61.3 mL (n = 116)	61.3 mL < RA-V_max_ (n = 32)	RA-V_preA_ < 22.5 mL (n = 27)	22.5 mL ≤ RA-V_preA_ ≤ 44.9 mL (n = 130)	44.9 mL < RA-V_preA_ (n = 22)	RA-V_min_ < 16.4 mL (n = 27)	16.4 mL ≤ RA-V_min_ ≤ 36.0 mL (n = 122)	36.0 mL < RA-V_min_ (n = 30)
V_max_ (mL)	32.6 ± 11.9	40.0 ± 10.6 ^†^	50.5 ± 15.0 ^†‡^	29.9 ± 8.6	41.0 ± 11.7 ^†^	52.2 ± 15.0 ^†‡^	31.7 ± 9.8	40.2 ± 11.5 ^†^	50.8 ± 14.8 ^†‡^
V_preA_ (mL)	21.5 ± 11.1	27.0 ± 9.9 ^†^	34.7 ± 14.5 ^†‡^	17.2 ± 6.5	27.9 ± 10.4 ^†^	39.0 ± 14.5 ^†‡^	18.5 ± 7.0	27.0 ± 10.0 ^†^	38.6 ± 14.3 ^†‡^
V_min_ (mL)	15.0 ± 6.8	19.3 ± 7.4 ^†^	24.5 ± 9.5 ^†‡^	12.2 ± 4.0	19.7 ± 7.3 ^†^	27.8 ± 9.5 ^†‡^	12.5 ± 4.7	19.2 ± 7.1 ^†^	27.2 ± 9.0 ^†‡^
TASV (mL)	17.6 ± 6.9	20.7 ± 7.3 ^†^	26.1 ± 8.5 ^†‡^	17.7 ± 7.4	21.3 ± 7.8 ^†^	24.4 ± 8.3 ^†^	19.1 ± 7.5	21.0 ± 7.7	23.6 ± 9.0 ^†^
TAEF (%)	54.1 ± 11.2	51.8 ± 12.7	52.1 ± 10.3	57.8 ± 12.1	51.9 ± 11.9 ^†^	46.9 ± 9.3 ^†‡^	59.5 ± 11.3	52.0 ± 11.6 ^†^	46.1 ± 10.6 ^†‡^
PASV (mL)	11.1 ± 4.2	12.9 ± 5.3	15.9 ± 6.6 ^†‡^	12.7 ± 5.5	13.1 ± 5.5	13.3 ± 5.8	13.2 ± 5.7	13.3 ± 5.4	12.2 ± 5.9
PAEF (%)	36.0 ± 12.5	33.0 ± 12.9	33.0 ± 12.8	42.2 ± 12.4	32.7 ± 12.3 ^†^	26.5 ± 11.0 ^†‡^	41.5 ± 11.2	33.6 ± 12.3 ^†^	25.0 ± 11.5 ^†‡^
AASV (mL)	6.5 ± 5.0	7.7 ± 4.9	10.2 ± 6.6 ^†‡^	5.0 ± 3.6	8.2 ± 5.4 ^†^	11.2 ± 6.2 ^†‡^	5.9 ± 3.6	7.7 ± 5.1	11.4 ± 7.2 ^†‡^
AAEF (%)	28.2 ± 11.3	28.0 ± 12.2	28.1 ± 10.1	27.3 ± 12.0	28.4 ± 12.1	27.5 ± 8.9	31.1 ± 11.0	27.5 ± 12.1	27.8 ± 10.2

Abbreviations: V_max_ = end-systolic maximum volume, V_preA_ = early diastolic preatrial contraction volume, V_min_ = late diastolic minimum volume, TASV = total atrial stroke volume, TAEF = total atrial emptying fraction, PASV = passive atrial stroke volume, PAEF = passive atrial emptying fraction, AASV = active atrial stroke volume, AAEF = active atrial emptying fraction, RA = right atrial, LA = left atrial. ^†^ *p* < 0.05 vs. lower than mean counterpart; ^‡^ *p* < 0.05 vs. mean counterpart.

**Table 6:** Intra- and interobserver variability for left and right atrial volumes as assessed by three-dimensional speckle-tracking echocardiography.

	Intraobserver Agreement		Interobserver Agreement	
	Mean ± 2 SD Difference in Values Obtained by two Measurements of the Same Observer	ICC between Measurements of the Same Observer	Mean ± 2 SD Difference in Values Obtained by two Observers	ICC between Independent Measurements of two Observers
LA-V_max_	0.4 ± 3.4 mL	0.96 (*p* < 0.001)	0.4 ± 4.9 mL	0.96 (*p* < 0.001)
LA-V_preA_	0.4 ± 3.4 mL	0.97 (*p* < 0.001)	0.4 ± 4.0 mL	0.97 (*p* < 0.001)
LA-V_min_	−1.1 ± 5.1 mL	0.87 (*p* < 0.001)	−1.0 ± 4.6 mL	0.88 (*p* < 0.001)
RA-V_max_	1.1 ± 5.9 mL	0.95 (*p* < 0.001)	1.1 ± 5.4 mL	0.97 (*p* < 0.001)
RA-V_preA_	−1.6 ± 7.8 mL	0.88 (*p* < 0.001)	−1.5 ± 7.9 mL	0.91 (*p* < 0.001)
RA-V_min_	0.9 ± 4.8 mL	0.93 (*p* < 0.001)	0.9 ± 4.2 mL	0.95 (*p* < 0.001)

Abbreviations. ICC = interclass correlation coefficient, SD = standard deviation, V_max_ = maximum end-systolic right atrial volume, V_preA_ = early diastolic pre-atrial contraction right atrial volume, V_min_ = minimum end-diastolic right atrial volume.

## Data Availability

Data available on request due to restrictions, e.g., privacy or ethical restrictions.
